# *Enterococcus innesii* sp. nov., isolated from the wax moth *Galleria mellonella*

**DOI:** 10.1099/ijsem.0.005168

**Published:** 2021-12-17

**Authors:** Harriet C. C. Gooch, Raymond Kiu, Steven Rudder, David J. Baker, Lindsay J. Hall, Anthony Maxwell

**Affiliations:** ^1^​ Dept. Biochemisty & Metabolism, John Innes Centre, Norwich Research Park, Norwich, NR4 7UH, UK; ^2^​ Quadram Institute Bioscience, Norwich Research Park, Norwich, NR4 7UQ, UK; ^3^​ Norwich Medical School, University of East Anglia, Norwich Research Park, Norwich, NR4 7TJ, UK; ^4^​ School of Life Sciences, ZIEL – Institute for Food &Health, Technical University of Munich, Freising, 85354, Germany

**Keywords:** *Enterococcus*, novel species, *Galleria mellonella*, wax moth, antibiotic resistance, vancomycin resistant

## Abstract

Four bacterial strains were isolated from two different colony sources of the wax moth *Galleria mellonella*. They were characterized by a polyphasic approach including 16S rRNA gene sequence analysis, core-genome analysis, average nucleotide identity (ANI) analysis, digital DNA–DNA hybridization (dDDH), determination of G+C content, screening of antibiotic resistance genes, and various phenotypic analyses. Initial analysis of 16S rRNA gene sequence identities indicated that strain GAL7^T^ was potentially very closely related to *Enterococcus casseliflavus* and *Enterococcus gallinarum *, having 99.5–99.9 % sequence similarity. However, further analysis of whole genome sequences revealed a genome size of 3.69 Mb, DNA G+C content of 42.35 mol%, and low dDDH and ANI values between the genomes of strain GAL7^T^ and closest phylogenetic relative *E. casseliflavus* NBRC 100478^T^ of 59.0 and 94.5 %, respectively, indicating identification of a putative new *Enterococcus* species. In addition, all novel strains encoded the atypical vancomycin-resistance gene *vanC-4*. Results of phylogenomic, physiological and phenotypic characterization confirmed that strain GAL7^T^ represented a novel species within the genus *Enterococcus *, for which the name *Enterococcus innesii* sp. nov. is proposed. The type strain is GAL7^T^ (=DSM 112306^T^=NCTC 14608^T^).

## Introduction

Enterococci are Gram-positive facultative anaerobes that are often diplococci, and which belong to the phylum *Firmicutes *, class *Bacilli *, order *Lactobacillales* and family *Enterococcaceae* [1, 2]. They comprise a large genus of lactic acid bacteria that are tolerant to many stress conditions and can be found in a wide range of habitats including water (fresh and marine), soils, and as members of animal, human and plant microbial communities (i.e. microbiomes) [3]. From a clinical perspective, some species, such as *Enterococcus faecalis* and *Enterococcus faecium *, are associated with opportunistic infections, including bacteraemia, endocarditis and urinary tract and catheter infections [4–6]. Crucially, *Enterococcus* species have inherent resistance to many antimicrobial agents including cephaloporins and β-lactams [7, 8]. They are also of further concern due to acquisition of multi-drug resistance traits, particularly rising rates of vancomycin-resistant *Enterococcus* strains [9], which are an increasingly common cause of infection in hospitals [10].

As highlighted above, *Enterococcus* species are also common animal microbiota members, and previous work has indicated that the greater wax moth, *Galleria mellonella*, is dominated by Enterococci [11, 12], like many other species of *Lepidoptera* [13]. Although *Galleria* is a pest of honeybee (*Apis mellifera*) hives worldwide [14], in recent years it has gained popularity as a model host for a range of human pathogens. It has the advantages of being inexpensive, easy to use, and able to grow at 37 °C, while not being subject to the same regulations and ethical concerns as mammalian models such as mice [15–17]. It has also been of interest due to the ability of the larvae to metabolize polyethylene [18]. Previous research on endogenous *Galleria* and *Enterococcus* species indicates these bacteria may have a colonization-resistance function, either passively or actively, through the production of antimicrobial bacteriocins [11, 19].

In this study, we isolated four bacterial strains initially identified as *Enterococcus casseliflavus* based on 16S rRNA gene alignments. However, on further inspection and characterization (genomic and phenotypic) we propose a novel and putative *Enterococcus* species: herein named *Enterococcus innesii* sp. nov. These data expand our knowledge of an important model organism-associated *Enterococcus* species, which encodes atypical vancomycin resistance genes and is therefore also of clinical importance.

## Isolation and ecology

*Galleria mellonella* larvae were obtained from a colony grown from larvae originally sourced from Livefood UK Ltd and maintained at the John Innes Centre Entomology Facility (Norwich, UK). *Galleria* larvae (TruLarv) were also purchased from BioSystems Technology. Larvae were flash-frozen in liquid nitrogen, and whole guts dissected under sterile conditions (three guts were pooled into each single sample). Each sample was then homogenized in 200 µl PBS, diluted 100-fold in PBS and 50 µl spread-plated on brain heart infusion (BHI) agar plates and incubated at 37 °C for 48 h. Individual bacterial colonies were selected and grown in BHI media. Three pure isolates from distinct single colonies were taken from *Galleria mellonella* larvae from the John Innes Centre Entomology Facility, and one was isolated from TruLarv larvae.

## Genomic characterization

The genomes of *E. innesii* GAL7^T^, *E. innesii* GAL9, *E. innesii* GAL10 and *E. innesii* TL2 were sequenced using the Nanopore MinION sequencing platform. Prior to this, FastDNA Spin Kit for Soil (MP Biomedicals) was used to extract genomic DNA from each isolate (grown up in BHI media for 48 h) following manufacturer’s instructions, with an extended 3 min bead-beating procedure as described previously [20]. The sequencing library was prepared via a modified Illumina Nextera Flex low input tagmentation approach using symmetrical 24 base barcoded primers [21]. Libraries were pooled and stringently size selected on a sageELF 0.75 % cassette and fractions from 4 kb and above were pooled and put into a standard Nanopore Ligation reaction using the SQK-LSK109 kit and protocol and loaded onto a MinION following the recommended loading guidelines and run for 48 h. Basecalling was performed using Guppy version 3.6.0 (Oxford Nanopore Technologies) in high accuracy mode (model dna_r9.4.1_450bps_hac). Subsequently, high-quality pure culture genomes (genome size range: 3.6–3.8 Mb) were assembled via Unicycler version 0.4.9 [22] and further polished using Racon version 1.3.1 in the Unicycler pipeline, with a range of 13–18 in contigs and G+C content of ~42 mol% (Table 1). Genomes were further annotated using Prokka version 1.13, with ~3800–4100 CDS predicted for these for *E. innesii* strains.

**Table 1.** Genome statistics comparison between closely related *Enterococcus* species (*n*=10) to *E. innesii* strains identified by TYGS, including type strain GAL7^T^ [45] Previously published type strain genomes were retrieved from GenBank for analysis in this study [46]. Genome assembly statistics were extracted using sequence-stats version 0.1 [47] while genome annotation was performed using Prokka version 1.13 [48].

Strains	Genome size (bp)	Contigs	G+C (mol%)	rRNA	tRNA	CDS	GenBank accessions
* Enterococcus alcedinis* CCM8433^T^	2 686 367	29	37.59	2	50	2472	GCA_014635985
* Enterococcus casseliflavus* NBRC100478^T^	3 498 264	54	42.35	3	50	3339	GCA_001544095
* Enterococcus devriesei* DSM22802^T^	3 320 653	65	40.22	1	29	3119	GCA_001885905
* Enterococcus gallinarum* NBRC100675^T^	3 774 884	87	39.75	3	49	3600	GCA_001544275
* Enterococcus gilvus* BAA350^T^	4 179 913	5	41.41	21	70	4111	GCA_000407545
*Enterococcus innesii* GAL10	3 678 879	18	42.32	15	69	3868	GCA_018982735
***Enterococcus innesii* GAL7 ^T^**	3 692 254	14	42.35	22	67	3866	GCA_018982785
*Enterococcus innesii* GAL9	3 793 471	13	42.22	18	64	4070	GCA_018982775
*Enterococcus innesii* TL2	3 806 372	17	42.25	20	63	4075	GCA_018982725
* Enterococcus malodoratus* ATCC43197^T^	4 654 237	10	39.56	16	54	4480	GCA_000407185
*Enterococcus massiliensis* AM1^T^	2 712 841	7	39.64	9	61	2612	GCA_001050095
* Enterococcus pseudoavium* NBRC100491^T^	2 731 874	59	40.06	3	48	2587	GCA_001544295
* Enterococcus saccharolyticus* ATCC13076^T^	2 604 038	2	36.70	6	38	2586	GCA_000407285
* Enterococcus viikkiensis* LMG26075^T^	2 545 311	45	40.26	4	40	2416	GCA_005405345

**Fig. 1.**
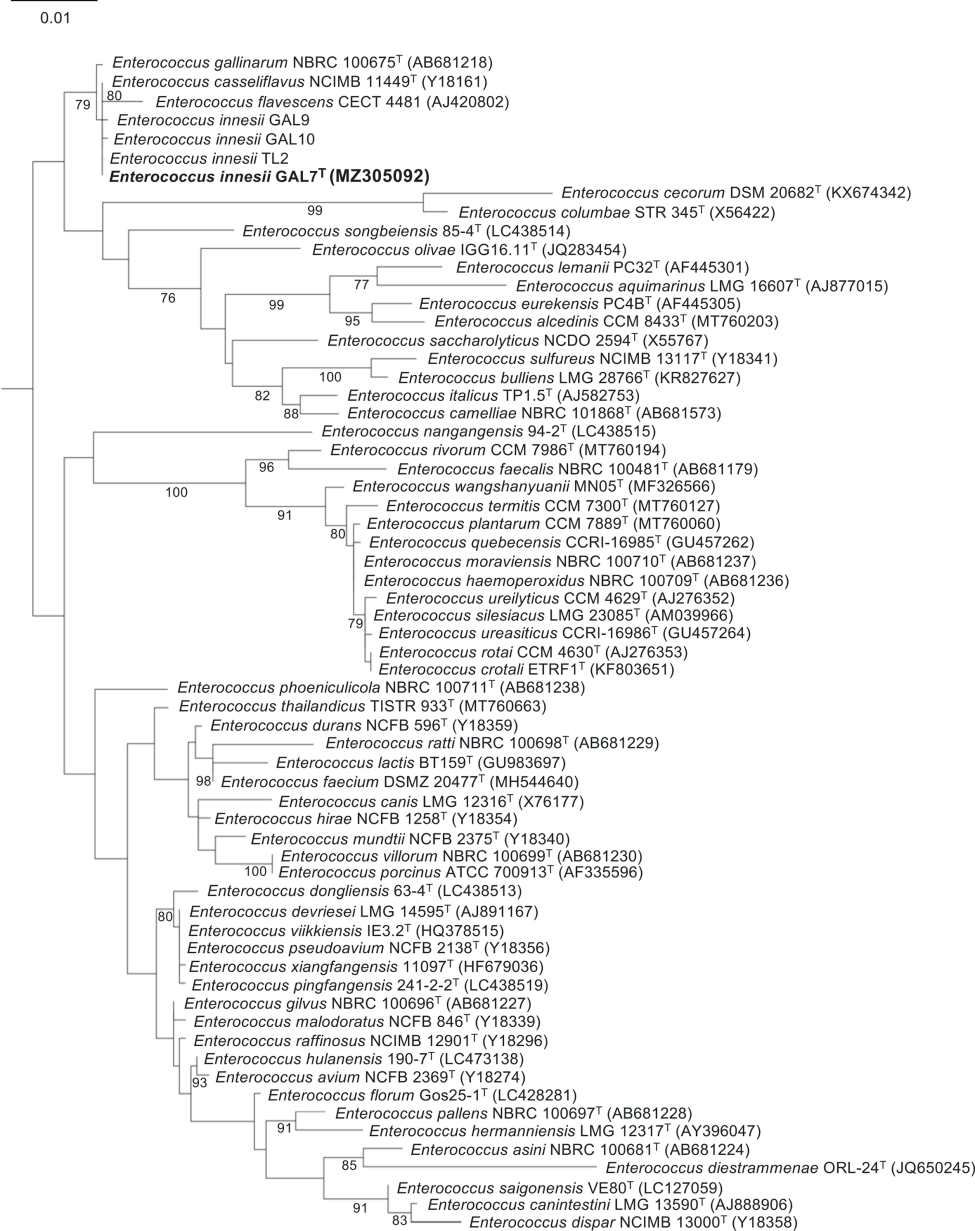
A mid-point rooted maximum-likelihood phylogenetic tree showing the phylogenetic position of *Enterococcus innesii* sp. nov. strain GAL7^T^ based on 16S rRNA gene sequences of 61 *Enterococcus* type strains. Bootstrap values (>70 %) based on 1000 replications are listed as percentages at the branches. Bar, 0.01 substitutions per nucleotide base.

**Fig. 2.**
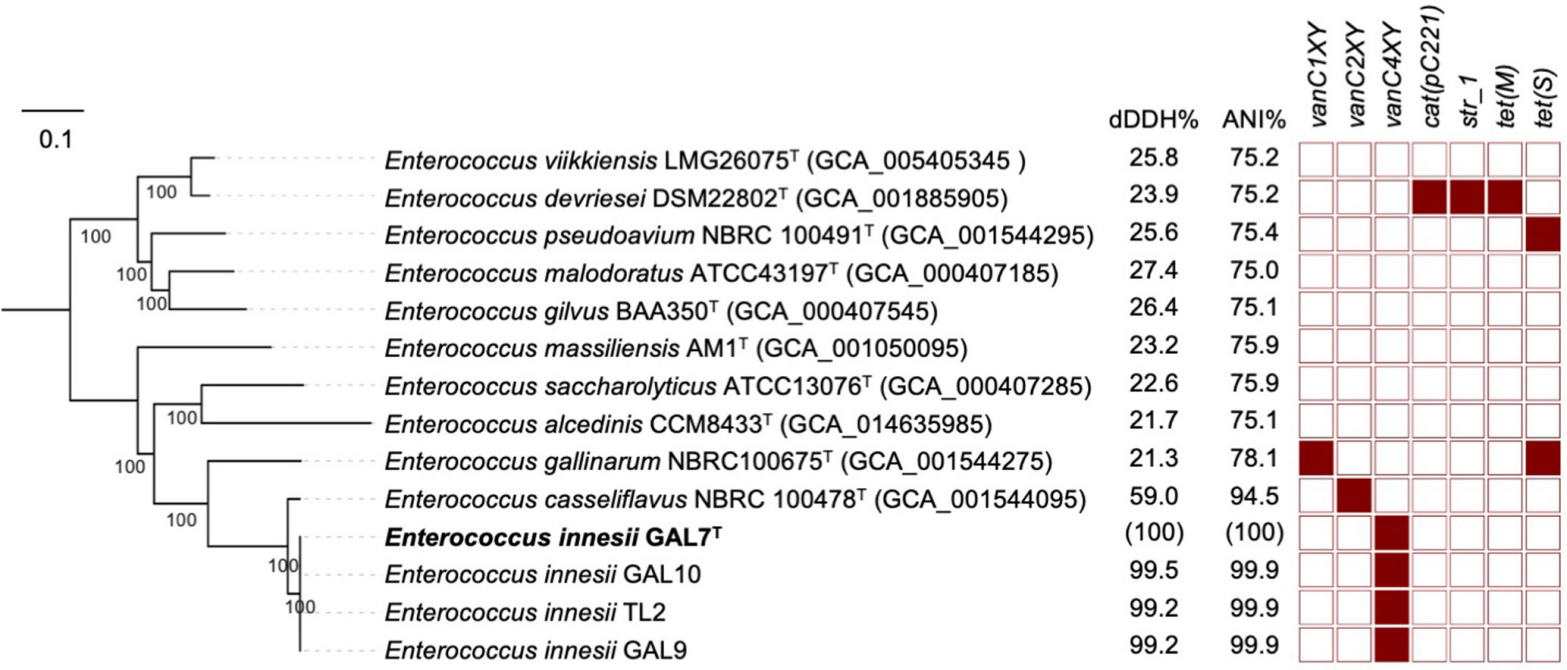
A mid-point rooted maximum-likelihood phylogenetic tree based on 154 826 single nucleotide polymorphisms from 564 core genes, aligned with dDDH (%), ANI (%) and antibiotic resistance gene profiles.

Initially, the 16S rRNA sequences of 61 validated *Enterococcus* species (60 were *Enterococcus* type strains) were obtained from the web server of List of Prokaryotic names with Standing in Nomenclature (LPSN; May 2021) [23, 24]. Using *in silico* approaches, near-full-length 16S rRNA sequences (~1.5 kb) of *E. innesii* were extracted via bactspeciesID version 1.2 [25], aligned with 16S rRNA sequences of other 61 public genomes using muscle version 3.8.31 [26], and a 16S rRNA-based maximum-likelihood phylogenetic tree was reconstructed via iq-tree version 2.0.5 with the GTR model at 1000 bootstrap replications while visualized with iTOL version 6 (Fig. 1) [27, 28]. *E. innesii* GAL7^T^ was phylogenetically positioned among *E. casseliflavus *, *E. flavescens* (re-classified as *E. casseliflavus *) and *E. gallinarum* cluster due to its 16S rRNA sequence similarity (99.53–99.93 %) [29]. However, when we compared the digital DNA–DNA hybridization (dDDH; via the Type Strain Genome Server, TYGS) and average nucleotide identity (ANI) for genome-based species delineation purposes (via fastANI v1.3), the proposed *E. innesii* sp. nov GAL7^T^ represented a separate species from *E. casseliflavus* and *E. gallinarum* type strains. The dDDH was 59.0 % (using TYGS formula d_4_) and ANI 94.5 %, when compared to its closest neighbour *E. casseliflavus* NBRC100478^T^, despite the high similarity of 16S rRNA sequences between the two species, both fell below the intra-species thresholds of 70 % dDDH and 95 % ANI (Fig. 2). In contrast, the ANI values among *E. innesii* strains (*n*=4) were 99.92–99.96 %.

Next, 10 closest-related *Enterococcus* strains (vs *E. innesii*) identified by TYGS were further examined phylogenetically at a genomic level, with antibiotic resistance genes also screened (using the *resfinder* database), for the four novel *E. innesii* strains (Fig. 2) [30]. The pangenome of these 14 strains were investigated using Roary version 3.12.0 [31] at blastp threshold at 70 % identity for inference of core genes. A total of 15 629 genes were present in this pangenome with 564 core genes and 15 065 accessory genes. Next, a core-gene alignment was generated and used to build a core-genome maximum-likelihood phylogenetic tree where it showed that *E. casseliflavus* NBRC100478^T^ was genomically distinct from *E. innesii*, further supported by single nucleotide polymorphism (SNP) analysis (using snp-dists version 0.7.0) that confirmed the SNP range (8–32 SNPs) among *E. innesii strains* (*n*=4) indicating strain distinction yet close genetic relatedness, while 11538–11540 SNPs were found when comparing *E. innesii* strains (*n*=4) and *E. casseliflavus* NBRC100478^T^ (Fig. 2) [32].

The vancomycin-resistance gene *vanC-4* (NCBI accession: EU151752) was uniquely detected (nucleotide sequence identity: 98.52–98.58 % at near 100 % coverage) in all *E. innesii* strains using ABRicate version 1.0.1 with the *resfinder* database, which was not found in any other closely related *Enterococcus* type strains (Fig. 2) [30, 33]. Notably, we did not detect any other virulence or antibiotic resistance genes in any of the four *E. innesii* strains. Vancomycin resistant determinant *vanC* subtypes had been reported in *E. gallinarum *, (*vanC-1*), *E. casseliflavus* (*vanC-2*), and *E. flavescens* (*vanC-3; E. flavescens* has now been re-classified as *E. casseliflavus *), while *vanC-4* has only been reported once previously in *E. casseliflavus *. In this study, the authors described the *vanC-4* encoding clinically associated *E. casseliflavus* isolates as having ‘at least two genetic lineages with the distinct *vanC* genes, that is, a single subtype including previously known *vanC-2/C-3*, and a novel subtype *vanC-4*′. We therefore propose that this distinct ‘genetic lineage’ of *E. casseliflavus* may hypothetically be *E. innesii*, a novel species that uniquely encode *vanC-4* gene [34, 35]. However, as these isolates described in this previous clinical study were not whole genome sequenced, we are unable to determine this conclusively. Furthermore, the *vanC* resistance gene was phenotypically demonstrated in *E. casseliflavus* and *E. gallinarum* as having intrinsic but low-level resistance to vancomycin at a minimum inhibitory concentration (MIC) of 4–32 µg ml^−1^ [36].

Subsequently, we screened through a larger public dataset of *Enterococcus* species via a targeted approach and found that three isolates previously designated as *E. casseliflavus* and *E. gallinarum* appeared to be *E. innesii* based on ANI (however, taxonomy check on NCBI were inconclusive for these isolates). These include *E. casseliflavus* NCTC4725 (ANI vs *E. casseliflavus* NBRC100478^T^: 94.88 %; ANI vs *E. innesii* GAL7^T^: 97.02 %), *E. gallinarum* FDAARGOS163 (ANI vs *E. gallinarum* NBRC100675^T^: 77.99 %; ANI vs *E. casseliflavus* NBRC100478^T^: 94.79 %; ANI vs *E. innesii* GAL7^T^: 95.40 %) and *E. gallinaru* m 4928STDY7071463 (ANI vs *E. gallinarum* NBRC100675^T^: 78.08 %; ANI vs *E. casseliflavus* NBRC100478^T^: 94.96 %; ANI vs *E. innesii* GAL7^T^: 95.43%). Importantly, these three isolates NCTC4725 (ATCC27284; GCA_901542395.1), FDAARGOS163 (GCA_001558875.2) and 4928STDY7071463 (GCA_902159265.1) are derived from human sources [37–39]. These isolates also demonstrated similar genome features as *E. innesii* sp. nov., with genome size range ~3.6–3.7 Mb and G+C ~42 mol%. These data suggest *E. innesii* sp. nov., may also be a clinically important species associated with novel antimicrobial resistance determinants, as *vanC-4* is encoded in all these genomes, and is reported to cause opportunistic human infection.

## Phenotypic characterization

Phenotypic characteristics were also investigated and included cell and colony morphology, motility, Gram-staining reaction, formation of endospores, oxygen relationship, growth at different temperatures, fermentation profiles of carbohydrates, catalase activity, oxidase activity, tolerance to NaCl, Voges–Proskauer reaction, urease production, pyrrolidonyl arylamidase production, hydrolysis of hippurate, deamination of arginine, pyruvate utilization, bile-aesculin tolerance test, haemolysis test, fatty acid analysis and vancomycin susceptibility testing [40]. Motility tests were carried out on *E. innesii* GAL7^T^ using motility test medium (Merck). Media were prepared according to manufacturer’s instructions and outcomes were recorded after culturing for 48 h at 37 °C. The susceptibility of *E. innesii* GAL7^T^ to antibiotic vancomycin was evaluated using MIC assays on BHI agar plates (carried out in three biological replicates) as described previously [41]. Aside from motility and vancomycin susceptibility tests, all phenotypic analyses were carried out by the Identification Service, Leibniz Institute DSMZ (Germany).

**Fig. 3.**
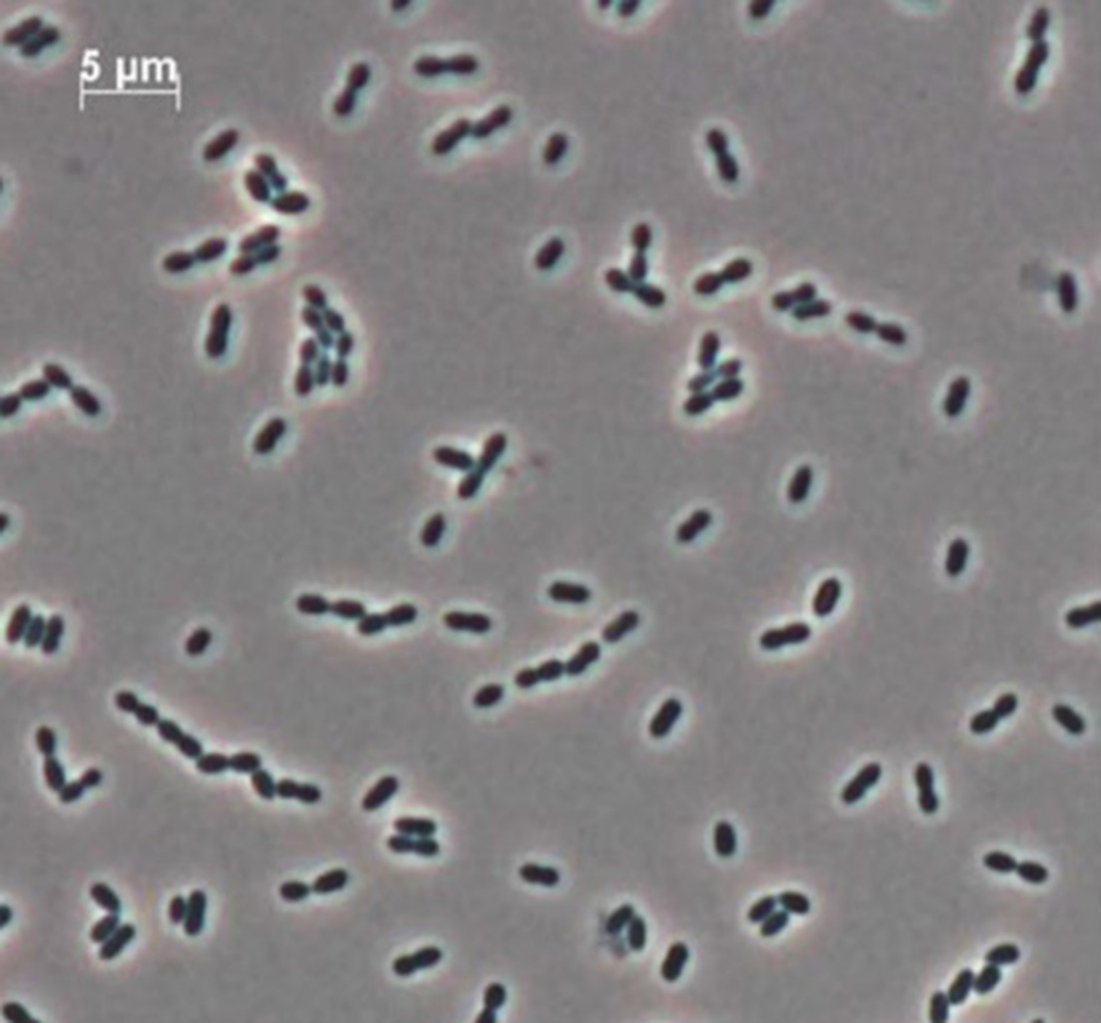
Phase-contrast microscopy showing *E. innesii* GAL7^T^ occurring in pairs and in chains.

*E. innesii* cells were coccoid-shaped, 1.0–1.5 µm long, motile and occurred in pairs or in chains under phase-contrast microscopy (Fig. 3). All *E. innesii* strains were Gram-positive, asporogenous, and facultatively anaerobic. Biochemical characteristics were determined using API 50CHE strips for carbohydrate utilization profiles, after incubation for up to 48 h at 37 °C (Table 2). They were capable of growth at 10–45 °C with optimum at 30–37 °C in BHI broth, with only weak growth at 45 °C, and no growth at 5 °C for up to 13 days. Growth was observed at NaCl concentrations from 0 to 8 % (w/v), with optimum growth <6.5 %. All strains were catalase- and oxidase-negative and showed no haemolytic activity. When compared to the closest related species *E. casseliflavus* (based on 16S rRNA analysis), *E. innesii* strains exhibited a distinctive metabolism in producing acid from glycerol, sorbitol, raffinose and 2-ketoglyconate, while not producing acid from turanose (Table 2). Further phenotypic features were determined using the API rapidID32 STREP system on single strain *E. innesii* GAL7^T^ where cells were negative for urease production, hydrolysis of hippurate and pyruvate utilization (no detectable growth using sodium pyruvate as sole carbon source in mineral salt medium for 6 days at 37 °C), while positive for Voges–Proskauer reaction, pyrrolidonyl arylamidase production and arginine dihydrolase. GAL7^T^ cells tested positive for aesculin hydrolysis in complex medium (Bacto-Peptone, 1 g l^−1^ aesculin). Moreover, similar to *E. gallinarum *, GAL7^T^ cells were positive for β-glucuronidase while closest relative *E. casseliflavus *, and related species *E. faecalis* and *E. faecium* were all negative for this enzyme (Table 2).

**Table 2.** Distinctive phenotypic features between *E. innesii* strains (data from this study) and phylogenetically closely related *E. casseliflavus* [49] and *E. gallinarum* strains [49], also distantly related *E. faecalis* [49] and *E. faecium* strains [49] +, All strains positive; −, all strains negative; +(−), most strains positive; −(+), most strains negative; v, variable; +w, most strains weakly positive, none negative. All strains were positive for ribose, galactose, glucose, fructose, mannose, *N*-acetylglucosamine, amygdalin, arbutin, salicin, cellobiose, maltose, lactose, trehalose and gentibiose. All strains were negative for erythritol, d-arabinose, l-xylose, adonitol, methyl β-xyloside, sorbose, dulcitol, inositol, xylitol, lyxose, d-fucose, l-fucose, d-arabitol, l-arabitol and 5-keto-gluconate.

Characteristics	E. innesii* (*n*=4)	* E. casseliflavus* † (*n*=6)	* E. gallinarum* † (*n*=4)	* E. faecalis* † (*n*=6)	* E. faecium* † (*n*=5)
Acid production from:					
d-Xylose	+	+	+	−	−
Sucrose	+	+	+	+	v
Melibiose	+	+	+	−	v
Methyl α-glucoside	+	+	+	−	−
Melizitose	−	−	−	+(−)	−
Mannitol	+	+	+	+	+(−)
Inulin	+	+	+	−	−
Gluconate	+	+	+	+(−)	v
l-Arabinose	+	+	+	−	+
Glycerol	+w‡	−	+	+	+
Rhamnose	+	+(−)	−	v	−
Sorbitol	v	−	+	+(−)	−
Methyl α-d-mannoside	+	+(−)	−	−	−(+)
Raffinose	+	−	+	−	−
Glycogen	−	−	−(+)	−	−
Turanose	−	v	+	−	−
d-Tagatose	−	−	+	+	−
2-Keto-gluconate	+	−	−	v	−
Hydrolysis of:					
Aesculin	+§	+	+	+(−)	+
Hippurate	−§	−	+	+(−)	+
Presence of enzymes:					
Arginine dihydrolase	+§	+(−)	+	+	+
α-Galactosidase	+§	+	+	−	−
β-Galactosidase	+§	+	+	−	+
β-Glucuronidase	+§	−	+	−	−

*Determined with the API 50CH system.

†Determined with the API 50CHE system.

‡Shaded area represents distinctive phenotypic features between *E. innesii* strain(s) and closely related *E. casseliflavus* and *E. gallinarum* strains as determined by API systems.

§Determined with API rapid ID32 STREP system on a single strain GAL7^T^.

Cellular fatty acids were analysed after conversion into fatty acid methyl esters (FAMEs) using a modified protocol by Miller [42]. Mixtures of the FAMEs were then separated by gas chromatography and detected by a flame ionization detector using the Sherlock Microbial Identification System (midi) based on TSBA6 database. C_14 : 0_, C_16 : 0_ and C_18 : 1_ *ω*7*c* were the major fatty acids in *E. innesii* GAL7^T^. Compared to the closest phylogenetic neighbours *E. casseliflavus* and *E. gallinarum* type strains (JCM8723^T^ and JCM8728^T^, respectively), *E. innesii* GAL7^T^ cells have a significantly higher C_14 : 0_ fatty acid content at 26.12%, apparently distinctive from *E. casseliflavus* (7.5 %) and *E. gallinarum* (0.2 %) as described previously [43].

Importantly, we determined that *E. innesii* GAL7^T^, which harboured putative atypical vancomycin resistance gene *vanC-4*, reduced susceptibility to vancomycin at MIC 4 µg ml^−1^ (vancomycin clinical breakpoint for Enterococci is >4 µg ml^−1^). This is similar to the low-level vancomycin resistance reported previously in *E. casseliflavus* and *E. gallinarum *, strains that encode the *vanC* resistance gene [36, 44].

Based on the results of phylogenomic, physiological and biochemical studies presented above, strain GAL7^T^ is considered to represent a novel species of the genus *Enterococcus *, for which the name *Enterococcus innesii* sp. nov. is proposed.

## Description of *Enterococcus innesii* sp. nov.

*Enterococcus innesii* (in.ne´si.i. N.L. gen. n. *innesii*, pertaining to British philanthropist John Innes JP and the John Innes Centre, Norwich, UK, where this bacterium was isolated).

Description is based on a single strain. Cells are Gram-positive, facultatively anaerobic, motile, non-haemolytic, asporogenous, coccoid-shaped, 1.0–1.5 µm long and usually occur in pairs or in chains. It grows at temperatures between 10–45 °C (optimum, 30–37 °C), at NaCl concentrations from 0 to 8.0 % (optimum, 0–6.5 %, at 37 °C) in BHI medium. Colonies formed on BHI after incubation for 48 h at 37 °C are non-pigmented, circular, smooth, shiny, diameter 1–2 mm, with entire margins. Negative for urease production, hydrolysis of hippurate, pyruvate utilization and catalase and oxidase production. Positive for Voges–Proskauer reaction, pyrrolidonyl arylamidase production, hydrolysis of aesculin and arginine dihydrolase. Acid is produced from l-arabinose, ribose, d-xylose, galactose, glucose, fructose, mannose, rhamnose, methyl α-d-mannoside, methyl α-glucoside, *N*-acetylglucosamine, amygdalin, arbutin, aesculin, salicin, cellobiose, maltose, lactose, melibiose, sucrose, trehalose, inulin, raffinose, gentibiose, gluconate, 2-ketogluconate, starch and glycerol. Acid is not produced from erythritol, d-arabinose, l-xylose, adonitol, methyl β-d-xyloside, sorbose, dulcitol, inositol, melizitose, glycogen, xylitol, turanose, d-tagatose, d-fucose, l-fucose, d-arabitol, l-arabitol and 5-ketogluconate. Resistant to 4 µg ml^−1^ vancomycin. The major fatty acids are C_14 : 0_, C_16 : 0_ and C_18 : 1_ *ω*7*c*.

The type strain, GAL7^T^ (=DSM 112306^T^=NCTC 14608^T^), was isolated from the gut of a wax moth *Galleria mellonella* at John Innes Centre (Norwich, UK). The genome of the type strain is characterized by a size of 3.79 Mb and a G+C content of 42.22 mol%.

## References

[1] Teixeira LM, Merquior VLC, Filippis de, McKee M (2013). Molecular Typing in Bacterial Infections (Infectious Disease).

[2] Jett BD, Huycke MM, Gilmore MS (1994). Virulence of enterococci. Clin Microbiol Rev.

[3] Gilmore MS, Clewell DB, Courvalin P, Dunny GM, Murray BE (2002). The Enterococci: Pathogenesis, Molecular Biology, and Antibiotic Resistance.

[4] Ceci M, Delpech G, Sparo M, Mezzina V, Sánchez Bruni S (2015). Clinical and microbiological features of bacteremia caused by *Enterococcus faecalis*. J Infect Dev Ctries.

[5] Nigo M, Munita JM, Arias CA, Murray BE (2014). What’s new in the treatment of enterococcal endocarditis?. Curr Infect Dis Rep.

[6] Flores-Mireles AL, Walker JN, Caparon M, Hultgren SJ (2015). Urinary tract infections: epidemiology, mechanisms of infection and treatment options. Nat Rev Microbiol.

[7] Miller WR, Munita JM, Arias CA (2014). Mechanisms of antibiotic resistance in enterococci. Expert Rev Anti Infect Ther.

[8] Sifaoui F, Arthur M, Rice L, Gutmann L (2001). Role of penicillin-binding protein 5 in expression of ampicillin resistance and peptidoglycan structure in *Enterococcus faecium*. Antimicrob Agents Chemother.

[9] Fisher K, Phillips C (2009). The ecology, epidemiology and virulence of *Enterococcus*. Microbiology.

[10] Arias CA, Murray BE (2012). The rise of the *Enterococcus*: beyond vancomycin resistance. Nat Rev Microbiol.

[11] Jarosz J (1979). Gut flora of *Galleria mellonella* suppressing ingested bacteria. J Invertebr Pathol.

[12] Allonsius CN, Van Beeck W, De Boeck I, Wittouck S, Lebeer S (2019). The microbiome of the invertebrate model host *Galleria mellonella* is dominated by *Enterococcus*. Anim Microbiome.

[13] Duplouy A, Hornett EA (2018). Uncovering the hidden players in Lepidoptera biology: the heritable microbial endosymbionts. PeerJ.

[14] Kwadha CA, Ong’amo GO, Ndegwa PN, Raina SK, Fombong AT (2017). The biology and control of the greater wax moth, *Galleria mellonella*. Insects.

[15] Tsai CJ-Y, Loh JMS, Proft T (2016). *Galleria mellonella* infection models for the study of bacterial diseases and for antimicrobial drug testing. Virulence.

[16] Lange A, Schäfer A, Bender A, Steimle A, Beier S (2018). *Galleria mellonella*: a novel invertebrate model to distinguish intestinal symbionts from pathobionts. Front Immunol.

[17] Pereira MF, Rossi CC, da Silva GC, Rosa JN, Bazzolli DMS (2020). *Galleria mellonella* as an infection model: an in-depth look at why it works and practical considerations for successful application. Pathog Dis.

[18] LeMoine CM, Grove HC, Smith CM, Cassone BJ (2020). A very hungry caterpillar: polyethylene metabolism and lipid homeostasis in larvae of the greater wax moth (*Galleria mellonella*). Environ Sci Technol.

[19] Johnston PR, Rolff J (2015). Host and symbiont jointly control gut microbiota during complete metamorphosisSymbiont Jointly Control Gut Microbiota during Complete Metamorphosis. PLoS Pathog.

[20] Alcon-Giner C, Dalby MJ, Caim S, Ketskemety J, Shaw A (2020). Microbiota supplementation with bifidobacterium and *Lactobacillus* modifies the preterm infant gut microbiota and metabolome: an observational study. Cell Rep Med.

[21] Baker DJ, Aydin A, Le-Viet T, Kay GL, Rudder S (2021). CoronaHiT: high-throughput sequencing of SARS-CoV-2 genomes. Genome Med.

[22] Wick RR, Judd LM, Gorrie CL, Holt KE (2017). Unicycler: Resolving bacterial genome assemblies from short and long sequencing reads. PLoS Comput Biol.

[23] Parte AC (2014). LPSN--list of prokaryotic names with standing in nomenclature. Nucleic Acids Res.

[24] Parte AC, Sardà Carbasse J, Meier-Kolthoff JP, Reimer LC, Göker M (2020). List of Prokaryotic names with Standing in Nomenclature (LPSN) moves to the DSMZ. Int J Syst Evol Microbiol.

[25] Kiu R (2020). BACTspeciesID: identify microbial species and genome contamination using 16S rRNA gene approach. https://github.com/raymondkiu/bactspeciesID.

[26] Edgar RC (2004). MUSCLE: multiple sequence alignment with high accuracy and high throughput. Nucleic Acids Res.

[27] Letunic I, Bork P (2019). Interactive Tree Of Life (iTOL) v4: recent updates and new developments. Nucleic Acids Res.

[28] Minh BQ, Schmidt HA, Chernomor O, Schrempf D, Woodhams MD (2020). IQ-TREE 2: new models and efficient methods for phylogenetic inference in the genomic era. Mol Biol Evol.

[29] Naser SM, Vancanneyt M, Hoste B, Snauwaert C, Vandemeulebroecke K (2006). Reclassification of *Enterococcus flavescens* Pompei *et al.* 1992 as a later synonym of *Enterococcus casseliflavus* (ex Vaughan *et al.* 1979) Collins *et al.* 1984 and *Enterococcus saccharominimus* Vancanneyt *et al.* 2004 as a later synonym of *Enterococcus italicus* Fortina *et al.* 2004. Int J Syst Evol Microbiol.

[30] Bortolaia V, Kaas RS, Ruppe E, Roberts MC, Schwarz S (2020). ResFinder 4.0 for predictions of phenotypes from genotypes. J Antimicrob Chemother.

[31] Page AJ, Cummins CA, Hunt M, Wong VK, Reuter S (2015). Roary: rapid large-scale prokaryote pan genome analysis. Bioinformatics.

[32] Seemann T, Klotzl F, Page AJ (2018). snp-dists: Pairwise SNP distance matrix from a FASTA sequence alignment. https://github.com/tseemann/snp-dists.

[33] Seemann T (2018). ABRicate: Mass screening of contigs for antimicrobial and virulence genes. https://github.com/tseemann/abricate.

[34] Clark NC, Teixeira LM, Facklam RR, Tenover FC (1998). Detection and differentiation of vanC-1, vanC-2, and vanC-3 glycopeptide resistance genes in enterococci. J Clin Microbiol.

[35] Watanabe S, Kobayashi N, Quiñones D, Hayakawa S, Nagashima S (2009). Genetic diversity of the low-level vancomycin resistance gene vanC-2/vanC-3 and identification of a novel vanC subtype (vanC-4) in *Enterococcus casseliflavus*. Microb Drug Resist.

[36] Cetinkaya Y, Falk P, Mayhall CG (2000). Vancomycin-resistant enterococci. Clin Microbiol Rev.

[37] Collins MD, Farrow JAE, Jones D (1986). *Enterococcus mundtii* sp. nov. Int J Syst Bacteriol.

[38] Sichtig H, Minogue T, Yan Y, Stefan C, Hall A (2019). FDA-ARGOS is a database with public quality-controlled reference genomes for diagnostic use and regulatory science. Nat Commun.

[39] Shao Y, Forster SC, Tsaliki E, Vervier K, Strang A (2019). Stunted microbiota and opportunistic pathogen colonization in caesarean-section birth. Nature.

[40] Mattarelli P, Holzapfel W, Franz CMAP, Endo A, Felis GE (2014). Recommended minimal standards for description of new taxa of the genera *Bifidobacterium*, *Lactobacillus* and related genera. Int J Syst Evol Microbiol.

[41] Andrews JM (2001). Determination of minimum inhibitory concentrations. J Antimicrob Chemother.

[42] Miller LT (1982). Single derivatization method for routine analysis of bacterial whole-cell fatty acid methyl esters, including hydroxy acids. J Clin Microbiol.

[43] Li YQ, Gu CT (2019). *Enterococcus pingfangensis* sp. nov., *Enterococcus dongliensis* sp. nov., *Enterococcus hulanensis* sp. nov., *Enterococcus nangangensis* sp. nov. and *Enterococcus songbeiensis* sp. nov., isolated from Chinese traditional pickle juice. Int J Syst Evol Microbiol.

[44] Brown DFJ, Wootton M, Howe RA (2016). Antimicrobial susceptibility testing breakpoints and methods from BSAC to EUCAST. J Antimicrob Chemother.

[45] Meier-Kolthoff JP, Göker M (2019). TYGS is an automated high-throughput platform for state-of-the-art genome-based taxonomy. Nat Commun.

[46] Benson DA, Karsch-Mizrachi I, Lipman DJ, Ostell J, Sayers EW (2009). GenBank. Nucleic Acids Res.

[47] Kiu R (2020). Sequence-stats: generate sequence statistics from FASTA and FASTQ files. https://github.com/raymondkiu/sequence-stats.

[48] Seemann T (2014). Prokka: rapid prokaryotic genome annotation. Bioinformatics.

[49] Collins MD, Jones D, Farrow JAE, Kilpperbalz R, Schleifer KH (1984). *Enterococcus avium* nom. rev., comb. nov.; *E. casseliflavus* nom. rev., comb. nov.; *E. durans* nom. rev., comb. nov.; *E. gallinarum* comb. nov.; and *E. malodoratus* sp. nov.. Int J Syst Bacteriol.

